# Carotenoids in the Management of Glaucoma: A Systematic Review of the Evidence

**DOI:** 10.3390/nu13061949

**Published:** 2021-06-06

**Authors:** Drake W. Lem, Dennis L. Gierhart, Pinakin Gunvant Davey

**Affiliations:** 1 College of Optometry, Western University of Health Sciences, 309 E Second St, Pomona, CA 91766, USA; drake.lem@westernu.edu; 2 ZeaVision, LLC, Chesterfield, MO 630005, USA; dgierhart@zeavision.com

**Keywords:** carotenoids, macular pigment, macular pigment optical density, MPOD, lutein, zeaxanthin, *meso*-zeaxanthin, glaucoma, primary open-angle glaucoma, retinal neurodegeneration

## Abstract

Primary open-angle glaucoma (POAG) remains a leading cause of irreversible blindness globally. Recent evidence further substantiates sustained oxidative stress, and compromised antioxidant defenses are key drivers in the onset of glaucomatous neurodegeneration. Overwhelming oxidative injury is likely attributed to compounding mitochondrial dysfunction that worsens with age-related processes, causing aberrant formation of free radical species. Thus, a compromised systemic antioxidant capacity exacerbates further oxidative insult in glaucoma, leading to apoptosis, neuroinflammation, and subsequent tissue injury. The purpose of this systematic review is to investigate the neuroprotective benefits of the macular carotenoids lutein, zeaxanthin, and *meso*-zeaxanthin on glaucomatous neurodegeneration for the purpose of adjunctive nutraceutical treatment in glaucoma. A comprehensive literature search was conducted in three databases (PubMed, Cochrane Library, and Web of Science) and 20 records were identified for screening. Lutein demonstrated enhanced neuroprotection on retinal ganglion cell survival and preserved synaptic activity. In clinical studies, a protective trend was seen with greater dietary consumption of carotenoids and risk of glaucoma, while greater carotenoid levels in macular pigment were largely associated with improved visual performance in glaucomatous eyes. The data suggest that carotenoid vitamin therapy exerts synergic neuroprotective benefits and has the capacity to serve adjunctive therapy in the management of glaucoma.

## 1. Introduction

Glaucoma is an optic neuropathy that is characterized by progressive neurodegeneration of the inner retina, including the optic nerve head (ONH) and retinal nerve fiber layer (RNFL), resulting in loss of retinal ganglion cells (RGCs) and characteristic visual field defects [1,2,3]. It remains the leading cause of irreversible vision loss globally and the projected prevalence of glaucoma is expected to reach 112 million in 2040 [3,4]. In the United States, the National Eye Institute estimates that over 4 million adults will be affected by 2030 and anticipates that the total will exceed 6.3 million in 2050 [5]. Similarly, healthcare expenditures related to glaucoma in the United States have been appraised at USD $2.5 billion annually [6]. Owing to the aging global population, the prevalence of glaucoma is expected to continue to rise and will remain a major global health problem.

The most common form of glaucoma in the United States, and worldwide, is primary open-angle glaucoma (POAG) [1]. Intraocular pressure (IOP) remains the only established modifiable risk factor for the incidence and progression of glaucoma [7,8]. Non-modifiable risk factors include age (≥60 years of age), race/ethnicity (e.g., individuals of African, Hispanic, or Latino descent), family history of glaucoma, myopia, type 2 diabetes mellitus, and central corneal thickness [9,10,11,12,13]. Current therapeutic approaches are aimed at delaying the disease’s progression by lowering the IOP through medical or surgical interventions [14,15]. However, a significant portion of patients with POAG still incur progressive glaucomatous vision loss despite maintenance of IOP within the normal statistical range [15].

Although the etiopathogenesis of glaucoma is not fully understood, there is a growing and evidence-based consensus that mitochondrial dysfunction and redox imbalance are likely involved in IOP elevation and the onset of neurodegeneration in the retina. Several theories of IOP-induced injury, including biomechanical deformation of lamina cribrosa and vascular dysregulation, consider overwhelming propagation of free radicals to be a key factor in perpetuating loss of RGCs [16,17,18]. Thus, an important mechanism in glaucomatous injury likely involves a vicious cycle of sustained oxidative stress whereby inhibition of the endogenous antioxidant defense systems potentiates retinal neurodegeneration [19,20,21,22,23,24,25]. Moreover, the retina is known to be particularly vulnerable to oxidative injury and free radical formation, in part due to its extremely high metabolic activity and constant exposure to light [26,27,28], wherein susceptibility increases with senescence [29,30,31,32]. Given the importance of macular pigment for optimal visual performance and maintaining retinal health, herein we review the current evidence in the literature investigating the neuroprotective association between macular carotenoid levels and glaucomatous neurodegeneration.

The body’s inherent defense mechanisms against oxidative damage, involving the neutralization of free radical species, rely upon the interplay between both endogenous and exogenous antioxidants to maintain redox homeostasis [21,33]. In particular, antioxidants such as the macular carotenoids lutein, zeaxanthin, and *meso*-zeaxanthin possess significant antioxidant and anti-inflammatory effects in the retina [34,35,36]. Clinical benefits in visual performance associated with dietary carotenoid supplementation have been demonstrated in healthy adults [37,38,39,40], as well as similar neurodegenerative retinopathies, including age-related macular degeneration [41,42,43] and diabetic retinopathy [44,45]. Despite similarities in pathogenesis involving sustained oxidative damage in the retina, only a limited number of studies have investigated the relationship between dietary carotenoid intake and pathophysiology in glaucoma.

However, experimental models of glaucomatous injury indicate that the carotenoids lutein and zeaxanthin may play a neuroprotective role against glaucomatous injury in the retina [46,47,48,49,50,51]. Lutein treatment in murine models of ischemia–reperfusion (I/R) injury was found to be effective in protecting against measures of oxidative and nitrosative stress [46,47,49] and enhance endogenous levels of glutathione (GSH) activity in rat retina [47]. Preliminary findings indicate that carotenoids may exert a synergic neuroprotective effect, at least in part by improving ganglion cell survival in the inner retina [46,48] while limiting activation of apoptotic pathways induced by glaucomatous injury [46,48,49,50,51,52]. Interestingly, not only was lutein treatment found to enhance RGC survival but also exhibited improvements against purported mechanisms contributing to secondary neurodegeneration in glaucomatous pathology [3,51,52,53].

To date, a limited number of observational studies [54,55,56,57,58,59] and clinical trials [60,61,62] have directly investigated the association between macular pigment optical density levels and open-angle glaucoma. Generally, evidence from clinical studies suggests that MPOD levels are reduced in glaucomatous eyes [56] and may be further compromised in relation to disease severity, particularly among those with foveal involvement [57,58,59]. However, some observational studies were unable to confirm that depletion of MPOD levels is associated with the presence of glaucoma [54,55]. The difference in study findings thus mandates a need for a consensus and a summary-generating systematic review of the literature.

## 2. Pathophysiology of Primary Open-Angle Glaucoma

### 2.1. Clinical Features of Primary Open-Angle Glaucoma

The defining features of glaucomatous damage are largely characterized by distinct changes to the ONH and corresponding visual field defects in consequence of neurodegenerative thinning of the nerve fiber layers [1,3]. Biomechanical deformation of lamina cribrosa in response to IOP-related injury can be identified by excavation (or “cupping”) of the optic disc, evidenced by progressive narrowing of the neuroretinal rim, and likely represents the initial site of RGC injury occurring at the ONH. Furthermore, characteristic patterns of peripapillary RNFL loss with corresponding arcuate visual field defects are common in patients with glaucoma [3].

In open-angle glaucoma, the iridocorneal angle remains visibly open upon clinical examination by gonioscopy, allowing for drainage of aqueous humor through the trabecular meshwork outflow pathway [1]. However, there is often greater resistance to or internal blockage of the aqueous outflow through the trabecular meshwork, causing an IOP elevation. Although IOP remains a strong primary risk factor for glaucomatous damage to the optic nerve and visual field, increased IOP is not required for diagnosis and damage can occur at any level of eye pressure [1,3].

### 2.2. Pathogenic Mechanisms of Glaucomatous Neurodegeneration

Glaucomatous pathology shares distinct features with several neurodegenerative disorders of the central nervous system, largely driven by overwhelming propagation of free radical species from compounding mitochondrial dysfunction with age [30,63,64,65] and either a tissue perfusion insufficiency or a bioenergetic crisis caused by a disruption in the ATP supply [23,53,66,67,68,69,70,71,72]. Consequently, and perhaps by definition, the magnitude of glaucomatous damage and RGC death is likely dependent upon, and determined by, the systemic antioxidant capacity in addition to any factors that may influence the local ocular redox status, and not solely upon IOP-induced injury.

In this regard, proliferative oxidative stressors and inhibition of endogenous antioxidant defenses have largely been considered the engine of neurodegenerative onset [19,20,21,22,23,72,73,74]. Thus, the mitochondrial redox balance becomes the central battleground for cell survival and worsening mitochondrial dysfunction may further compromise RGC viability [16,31,65]. Sustained oxidative injury likely acts as the primary mechanism of glaucomatous tissue damage, wherein it ultimately leads to progressive loss of RGCs and subsequent functional deterioration of the optic nerve. Glaucomatous neurodegeneration occurs primarily by induction of programmed cell death, often proceeding through either intrinsic or extrinsic apoptotic pathways that rely upon caspase-dependent activity [31,65,75]. There is evidence of significant oxidative damage in the trabecular meshwork causing resistance to aqueous humor outflow and subsequently an IOP elevation in patients with POAG [29,76,77]. Furthermore, the multifactorial nature of glaucomatous neurodegeneration strongly indicates that several interconnected mechanisms are likely to contribute to RGC loss and subsequent visual field defects [2,3,53,78].

Emerging findings suggest that the glaucomatous retina may become an environment hostile to RGC survival, wherein pro-oxidant and pro-inflammatory processes perpetuate glaucomatous neurodegeneration over time. Persistent overwhelming oxidative insult can trigger immunostimulatory activation of the innate immune system, causing a concurrent low-grade inflammatory response in the retina involving resident immune cells [19,79,80]. For instance, immunostimulation of the inflammasome oligomerization through purinergic signaling via P2X7 receptor activation has been shown to play a key role in contributing to RGC toxicity and cell death [81]. Over time, the resultant parainflammatory response, or adaptive immune response to inflammatory stimuli in order to restore homeostatic processes [82], becomes dysregulated and advances into a state of chronic inflammation [19,79,80]. Thus, in response to further oxidative insult, the over-activated glial cells, namely microglia, release pro-inflammatory molecules such as tumor necrosis factor alpha (TNF-α), nitric oxide synthase (NOS), and cyclooxygenase-2 (COX-2) [79,80,83,84,85]. Retinal injury due to pro-oxidant and pro-inflammatory stressors induced by glaucomatous tissue injury may be further exacerbated by chronic activation of the innate immune system.

Glutamate neurotoxicity has been previously considered among several primary contributing factors sufficient to induce the death of ganglion cells and optic nerve damage in glaucoma. Theories regarding excitotoxicity as a causal mechanism in glaucomatous injury are largely based on seminal findings wherein elevated intraocular levels of the excitatory neurotransmitter were reportedly found in glaucomatous eyes [86,87]. However, there is a growing evidence-based consensus that is not in agreement with this hypothesis [87,88,89,90,91], and there is substantial evidence that intravitreal glutamate levels are not elevated in humans [89] or animal models of glaucoma [90,91]. Despite this, glutamate is an integral component of the mammalian central nervous system (CNS) and dysregulated excitatory neurotransmission can lead to proliferative excitotoxic cell death in neurons [92,93,94]. Given that the axons of RGCs are direct extensions of the CNS, glutamate neurotoxicity may still be relevant in glaucomatous pathology, likely contributing to progressive secondary neurodegeneration in response to overwhelming oxidative injury.

Glaucomatous optic neuropathy is marked by a substantial loss of RGCs and their axons, which suggests that progressive damage is not limited to retinal substructures or the optic nerve. Previous reports have corroborated the structure–function relationship between neurodegenerative RGC death and functional vision loss in glaucomatous eyes [95,96,97,98,99]. Correspondingly, the process of transsynaptic degeneration likely exacerbates axonal injury, in consequence of biomechanical stress or a vascular insufficiency, wherein the neurodegenerative cascade, which results in apoptotic death of RGCs, disseminates along the entire visual pathway [23,53,71,100,101,102,103]. There is a strong line of evidence whereby both Wallerian (anterograde) and retrograde degeneration can be attributed to axonal damage and loss of RGC somas in the human visual system [102]. In this regard, one may presume that for each ganglion cell in the retina, a corresponding neuron is present in the retinogeniculate pathway, and, therefore, a substantial loss of RGCs (i.e., visual field defects) may be indicative of significant neurodegenerative loss at the visual cortex level [104]. In fact, secondary degeneration was observed in the lateral geniculate nucleus (LGN) with corresponding damage to the ONH in primates following artificial IOP elevation [103]. Furthermore, structural magnetic resonance imaging (MRI) studies in patients with POAG have revealed significant neurodegenerative alterations in the LGN and central cortical structures of the visual pathway, as evidenced by shrinkage in the LGN height and volume after region of interest (ROI)-based analysis [105]. Similar neuroimaging studies reported a marked reduction in axonal volume in the optic radiations originating from the thalamus and primary visual cortex concomitant with atrophy of the LGN [100,105]. Thus, adjunctive therapeutic strategies must be aimed at simultaneously slowing down glaucomatous damage concomitant with exerting a neuroprotective effect on RGCs in glaucoma.

## 3. Macular Pigment Optical Density in the Management of Glaucoma

### 3.1. Role of Macular Pigment Optical Density

**Figure 1:**
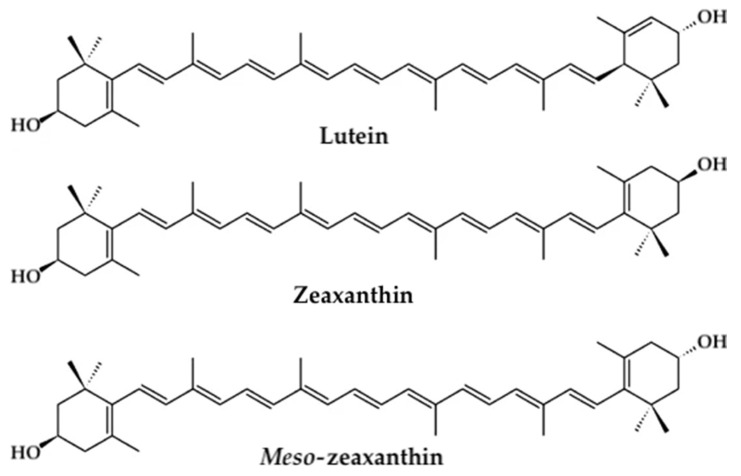
Chemical structures of the xanthophyll carotenoids lutein, zeaxanthin, and *meso*-zeaxanthin.

The xanthophyll carotenoids lutein, zeaxanthin, and *meso*-zeaxanthin play a crucial role in preserving retinal health while maintaining optimal visual acuity and central vision mediated by the macula (Figure 1) [34,106,107,108,109,110]. Collectively, these carotenoids constitute the macular pigment, wherein they are uniquely concentrated within the axons of photoreceptor cells and the inner plexiform layer and outer plexiform layer of the foveal center in the macular region [106,107,109,111,112]. Obtained exclusively from dietary intake, lutein and zeaxanthin cannot be synthesized in the body [107,113,114] and must be acquired from foods such as leafy green vegetables, corn, and egg yolks [107,110,113,114]. *Meso*-zeaxanthin is a metabolite of lutein’s transformation through RPE65 isomerase conversion in retinal pigment epithelium (RPE) [34,107,113,115,116,117,118,119,120,121,122]. Retinal uptake, metabolism, and transport mechanisms of xanthophyll carotenoids have been discussed in more detail elsewhere [34,107,113,116,117,119,121,122,123]. Depletion of these macular pigments, namely low macular pigment optical density (MPOD), may be associated with a significant increase in the risk of incident retinopathy and impaired visual function.

Dietary carotenoid supplementation may offer neuroprotection in the retina by augmenting the MPOD and subsequently delay the onset of glaucomatous pathology. The macular pigments are believed to protect the retinal tissue, in particular the photoreceptor cells located in the central region, through two primary mechanisms: (1) by acting as a filter against blue light; and (2) by limiting oxidative stress and inflammation induced by free radical species [107,108,110,124,125,126,127]. The peak wavelength of the MPOD absorption spectrum (~460 nm) enables macular pigment to absorb a range of visible blue light (400–500 nm), thereby reducing the exposure of photoreceptors to blue light concomitant with improvements in visual performance [106,126,128]. The enhancement in optical filtration is particularly important because short-wavelength (blue) light is of high energy with significant potential to exacerbate photo-oxidative injury and ROS production in the highly susceptible layers of the outer retina [107,125,126,127,128,129]. Moreover, a growing body of evidence strongly indicates that higher MPOD levels afford enhanced retinal protection against the onset of neurodegeneration contributing to several ocular diseases, including glaucoma [107,108,109,110].

### 3.2. Measuring MPOD

Currently, several techniques are used to measure MPOD; they offer unique advantages as well as clinical limitations that have been discussed more thoroughly elsewhere [34,109,110,130,131,132,133,134,135]. In summary, non-invasive methods for quantifying levels of macular pigment include heterochromatic flicker photometry (HFP), customized HFP (cHFP), fundus reflectometry, and autofluorescence. The most commonly used are the psychophysical techniques HFP and cHFP [34,109,110,134,135,136,137,138], which rely on subjective perceptions to estimate the level of MPOD [139,140,141]. Conversely, the fundus reflectometry [142,143,144,145,146,147] and autofluorescence imaging (AFI) [132,133,148,149] techniques rely on physical properties of the retina in order to collect measurements such as light reflectance and lipofuscin fluorescence, respectively [34,110,130,131,134,150,151]. It is important to note that validated measurements of MPOD levels can serve as susceptibility/risk biomarkers for the screening of early glaucomatous damage involving the macula [99], which may be clinically evident prior to central visual field loss on standard automated perimetry.

## 4. Materials and Methods

This systematic review was conducted in accordance with the Preferred Reporting Items for Systemic reviews and Meta-Analysis (PRISMA) reporting guidelines [152].

### 4.1. Literature Search

A comprehensive literature review was conducted to identify published articles on the topic using database searches from PubMed, Web of Science, and the Cochrane Library indexes. We retrieved all relevant publications that reported findings on the association between glaucoma and MPOD/carotenoids (lutein and/or zeaxanthin and/or *meso*-zeaxanthin) from clinical and pre-clinical studies prior to 10 March 2021. The database search keywords used in the query included a combination of the following and their variants: carotenoids, lutein, zeaxanthin, macular pigment, macular pigment optical density, MPOD, antioxidants, glaucoma, open-angle glaucoma, and glaucomatous neurodegeneration. Initial publication results were screened for appropriate selection criteria and reviewed further based on titles and abstracts available in English. From the eligible publications, we manually performed both backward and forward searches from the reference lists and cited references to include all relevant literature, respectfully. Two authors (PGD and DWL) individually screened all eligible full-text studies for the inclusion/exclusion criteria outlined below, and any discrepancies were resolved by discussion including the third author (DLG).

### 4.2. Selection Criteria

Preclinical studies that satisfied the following criteria were included in this review: (1) evaluated the effect of carotenoid (lutein and/or zeaxanthin and/or *meso*-zeaxanthin) treatment on retinal cell survival, oxidative stress measures, or neurodegeneration-mediated outcomes in glaucoma-related injury, through cell culture studies of hypoxia/ischemia or experimental animal models of ischemia–reperfusion (by artificial IOP elevation or a cerebral artery occlusion model); (2) analyzed carotenoid treatment separately from other treatments/antioxidants in comparison with controls; and (3) used a methodology that was pertinent to glaucomatous-related injury in humans.

Observational clinical studies in this review were required to adhere to the following criteria: (1) studies evaluating the association between risk of open-angle glaucoma and carotenoid levels through dietary consumption of lutein and/or zeaxanthin or by validated clinical measurement of macular pigment optical density (MPOD) levels; (2) studies involving adults with confirmed presence of open-angle glaucoma or POAG; and (3) peer-reviewed original research.

Prospective randomized clinical trials that satisfied the following criteria were included in this review: (1) interventional studies assessing the effects of nutraceutical carotenoid supplement, containing lutein and zeaxanthin, on clinical endpoints in glaucoma patients; (2) studies that included human adults with the presence of open-angle glaucoma; and (3) peer-reviewed original research.

### 4.3. Data Extraction and Reliability

The PRISMA reporting guidelines were followed with care as closely as possible, as described previously [152].

## 5. Results

### 5.1. Search and Selection of Studies

**Figure 2:**
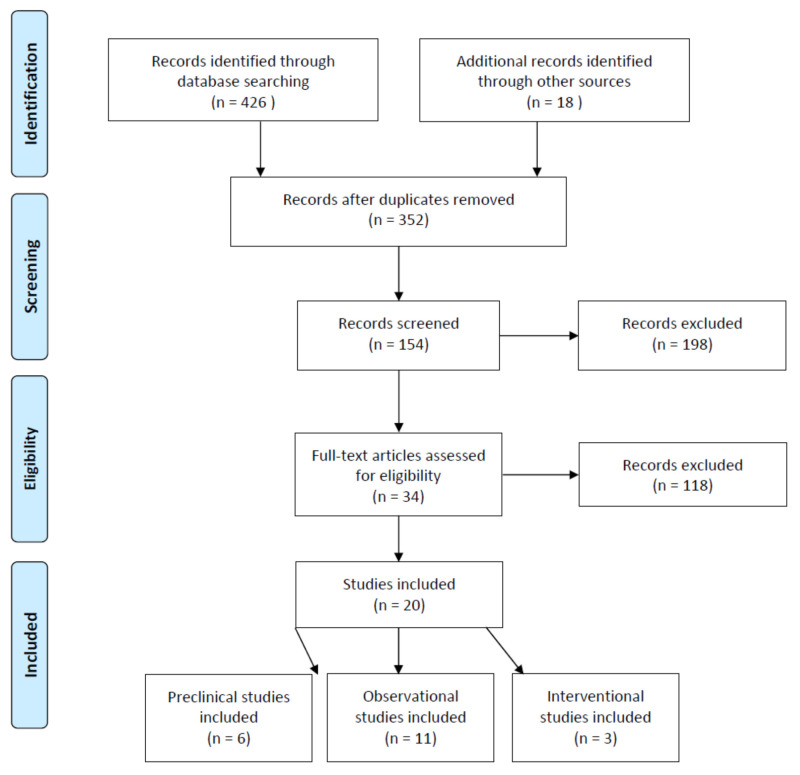
PRIMSA flow chart of the systematic review of publications on carotenoids in the management of glaucoma.

In total, 426 studies were identified during the initial search from scientific databases. After duplicate records were removed and additional records were retrieved from reference lists, 352 studies remained for title and abstract screening. From these 352 studies, 198 records were excluded based on article type. Consequently, 118 records were excluded due to the aforementioned inclusion criteria for preclinical and clinical studies, and 34 studies were identified to be eligible for full-text assessment. Finally, 14 records were excluded because of the methodology used, with a resulting 20 studies included in the final review. Of the 20 studies analyzed, six were preclinical studies [46,47,48,49,50,51], eleven were observational clinical studies [54,55,56,57,58,59,153,154,155,156,157], and three were randomized controlled trials [60,61,62] (Figure 2).

### 5.2. Carotenoids in the Management of Glaucoma (Preclinical Studies)

The therapeutic benefits of macular carotenoids have been documented in experimental models of glaucomatous pathology, investigating the molecular mechanisms underlying RGC loss; in particular, the protective effects of lutein and/or zeaxanthin on the progression of neurodegeneration in glaucoma (Table 1) [46,47,48,49,50,51]. Data from these reports are consistent with corroborating evidence that administration of the carotenoids lutein and zeaxanthin may provide substantial neuroprotective benefits in the retina by counteracting the causative factors that contribute to glaucomatous injury. Experimental models of glaucomatous neurodegeneration in vivo can be emulated in part by inducing retinal ischemia–reperfusion (I/R) via: (1) increasing IOP above systolic blood pressure by cannulation of the eye [46,47,158,159,160,161,162]; or (2) ligation of the internal carotid artery by an intraluminal method [49,50,159,162,163,164,165]. In rat Muller glial cells (rMC-1), in vitro hypoxic/ischemic conditions can be induced using cobalt (II) chloride (CoCl_2_) to generate ROS/oxidative stress resulting in cell death [48,50,166]. Experimental murine models of RGC dysfunction/loss can be simulated by intravitreal injection of N-methyl-d-aspartic acid (NMDA) to mimic the in vivo glutamate excitotoxicity, which is triggered by ligand binding of NMDA receptors expressed by these ganglion cells in retinal neurons [51,167].

Experimental models of glaucomatous neurodegeneration implicate oxidative stress among the primary mechanisms in early ischemic retinal injury, caused by overwhelming production of pro-oxidant stressors and a compromised antioxidant capacity. It is known that the retina is highly susceptible to free radical formation partly because of its extremely high metabolic activity and abundance of polyunsaturated fatty acids concentrated in the outer segment of photoreceptor cells [26,27,28]. In murine models of acute I/R, lutein’s neuroprotective effect was seen to successfully prevent against measures of both oxidative and nitrosative stress that have been attributed to glaucomatous neurodegeneration [46,47,49]. Carotenoids may protect the inner retina by actively neutralizing free radicals and concomitantly protecting against subsequent oxidative injury, such as lipid peroxidation and oxidative DNA damage [168,169]. Indeed, treatment with lutein effectively prevented increases in malondialdehyde [47] and polymeric adenosine diphosphate ribose (PAR) [49] induced by I/R injury, respectively. Lutein has also been observed to be neuroprotective against nitrosative stress in ischemic retina by reducing NOS activation and thereby limiting the subsequent overexpression of nitric oxide (NO), both of which are recognized to contribute significantly to neurodegeneration [46,49,170,171]. Evidence from murine models seems to mirror these findings, where it was observed that lutein can successfully diminish nitrosative injury as evidenced by reduced expression levels of neuronal NOS [46] and nitrotyrosine [49], a footprint indicator of protein oxidation by reactive nitrogen species [70,170,172]. The anti-oxidative capacity of macular carotenoids, particularly in ischemic retina, is important because both vascular and biomechanical theories of glaucoma attribute IOP-related injury to the ONH and progressive loss of RGCs exacerbated by oxidative stress [28,78,173,174].

Thus, a retinal imbalance between the overwhelming generation of pro-oxidant stressors and a compromised antioxidant capacity has been posited, among several mechanisms, to be an important causative agent of glaucomatous RGC loss. In humans, there is evidence of an intracellular redox imbalance whereby endogenous antioxidant defenses are compromised in consequence of uncontrolled oxidative stress [175,176]. However, there is a paucity of preclinical research investigating the antioxidant capacity in experimental models of glaucomatous-related injury [47]. In fact, only one study evaluated lutein treatment on concentrations of the endogenous antioxidant GSH in rat retina following I/R by artificial IOP elevation [47]. Administration of lutein was effective against depletion of GSH induced by ischemic injury and restored GSH to levels similar to those observed in healthy control animals. Alternatively, evidence from murine models of diabetic retinopathy helps to corroborate the synergic neuroprotective effect of carotenoid treatment (including lutein and/or zeaxanthin) on limiting oxidative stress while simultaneously augmenting endogenous antioxidant defenses [177,178,179].

Macular carotenoids may protect against glaucomatous injury indirectly by inhibiting pro-inflammatory pathways triggered by aberrant free radical production and oxidative insult contributing to chronic low-grade inflammation [50,180,181,182,183]. Lutein’s potent anti-inflammatory effects have been demonstrated to protect against post-ischemic injury via modulating activation of the nuclear transcription factor nuclear factor kappa B (NF-kB), a vital redox-sensitive transcriptional regulator of pro-inflammatory cytokines and secondary inflammatory markers expressed during the innate immune response [50,184,185,186,187,188,189,190,191]. Additionally, lutein treatment was also observed to suppress retinal expression of COX-2 [46,50] and interleukin-1β [50,192]. The former is a stress response gene [190] and the latter triggers the NF-kB canonical inflammatory response pathway upon cytokine receptor activation [189,191,193]. Similarly, treatment with lutein produced a marked decrease in Muller cell gliosis [50], a significant source of pro-inflammatory cytokine production [48,80,194,195]. In ischemic retinal injury, significant attenuation of Muller cell hypertrophy and glial fibrillary acidic protein activation were achieved following treatment with lutein [50]. Interestingly, these findings suggest that the neuroprotective mechanism may involve the adaptive injury response facilitated by reciprocal cell signaling between Muller cells and microglia during post-injury inflammation; in fact, activation of microglia and activation of macroglia (i.e., Muller cells and astrocytes) are among the initial steps in the neurodegenerative onset that precedes RGC loss in humans [196,197,198,199,200]. Thus, the retinal benefits of lutein may limit the propagation of immune response pathways to suppress neuroinflammation and, in effect, preserve the inner retina against subsequent RGC loss and apoptotic degeneration. 

The cumulative effect of glaucoma is progressive neurodegeneration of the inner retina and RGC loss by apoptotic mechanisms [72,201,202]. In experimental models of retinal injury by artificial IOP elevation [161,203] and an intraluminal method [49,50,204], significant cell loss was observed in the ganglion cell layer (GCL) and the inner nuclear layer (INL) as evidenced by loosely packed cells and condensation of nuclear chromatin. Following treatment with lutein, inner layers of mouse retina were seen to have densely packed cells and a marked reduction in pyknotic nuclei, indicating a normal morphology following ischemic injury [49,50]. Lutein-mediated protection of inner retinal cells may in part be explained by improvements observed in cell viability [48,49,50,51] and cell survival [46,48] upon glaucomatous injury. Administration of lutein was shown to increase cell survival in both the GCL and the INL of ischemic rat retina by IOP elevation [46] and enhance the survival of Muller glia after CoCl_2_-induced hypoxia [48]. Experimental findings suggest that lutein may protect the retinal tissue by augmenting cell survival concomitant with protecting against glaucomatous cell death induced by an apoptotic mechanism. Indeed, administration of lutein significantly reduced the presence of apoptotic nuclei in Muller glia upon a terminal deoxynucleotidyl transferase dUTP nick end labeling (TUNEL) assay following hypoxic injury by chemical induction [48]. Improvements were also observed in the GCL and the INL of mouse retina following intraluminal I/R injury [49], providing supplementary evidence that lutein may successfully protect against apoptotic neurodegeneration in the inner retina. Thus, preliminary findings indicate that the neuroprotective potential of lutein in maintaining the retina, an integral component of the central nervous system, is essential to preventing irreversible neural degeneration and subsequent vision loss.

Lutein may protect the inner retina against glaucomatous neurodegeneration by inhibiting pro-apoptotic pathways induced by hypoxic/ischemic injury. In fact, the anti-apoptotic potential of lutein has been documented in a variety of in vivo and in vitro experimental models of retinopathies characterized by neurodegeneration [48,50,52,182,205,206,207]. One study in Muller glia cells suggests that the possible mechanistic pathway of lutein’s protection involves suppression of the intrinsic mitochondrial pathway after simulated hypoxic challenge [48]. Lutein treatment effectively suppressed activation of the intrinsic apoptosis induced by hypoxic injury [208,209], as evidenced by increased expression of the pro-survival protein B cell lymphoma 2 (Bcl-2) and a reduced expression ratio of the Bax/Bcl-2 proteins [48], a key determinant in apoptosis triggered by mitochondrial dysfunction [30,210,211,212,213,214]. Importantly, lutein successfully reduced cleavage of caspase-3 [48], effectively limiting activation of the executioner caspase and supporting lutein-mediated neuroprotection through caspase-associated modulation [30,210,211,212,214,215]. Results in an animal model of ischemic injury mirrored these findings, where it was observed that lutein had a significant ameliorative effect on caspase-3 activation induced by elevated IOP in rat retina [47]. Lutein’s reducing of ischemic apoptosis is important because bi-directional signaling between microglia and Muller cells in response to injury [196,197] can exacerbate neurotoxic effects by enhancing oxidative stressors and further spreading neuroinflammatory processes via cytokine signaling [216,217,218,219,220].

Carotenoids have also been shown to be neuroprotective in maintaining synaptic activity. One school of thought suggests that the protective mechanism of carotenoids involves limiting trans-synaptic degeneration. That is, carotenoids limit secondary degeneration to neurons that follows remote neuronal injury, by supplying significant synaptic activity and, thereby, decreasing retinal apoptosis [53,71,102]. Purported mechanisms underlying cell death in RGCs are hypothesized to involve excitotoxicity resulting from aberrant glutamate receptor activation [221,222,223,224,225,226] or neurotrophic signaling deprivation caused by deficits in axonal transport [227,228,229]. One study found that lutein treatment significantly enhanced RGC viability in rat retina following NMDA-induced neurotoxicity [51]. In fact, a Western blot analysis of mitochondrial apoptotic proteins mirrored these findings, indicating that lutein rescued RGC viability by augmenting retinal expression of the pro-survival protein Bcl-2 while inhibiting the expression of Bax, cleaved caspase-3, and cytochrome c [51]. Lutein supplementation may enhance RGC survival in part by improving the axoplasmic flow of neurotrophins between the ganglion cell body and its distal synapse located deep in the brain [53,230]; in fact, lutein was seen to increase retrograde transport of brain-derived neuronal trophic factor (BDNF) in a murine model of diabetic neurodegeneration [205]. Based on observations made in experimental models, it has been hypothesized that the primary site of axonal transport dysregulation is the lamina cribrosa and it may be triggered by biomechanical stress/damage to the ONH and RNFL [53,227,230,231,232]. Thus, the benefits of lutein treatment observed in experimental models of glaucomatous neurodegeneration may in part be explained by augmentation of RGC survival and thereby preservation of synaptic network activity within retinogeniculate axons that relay visual information to the visual cortex [53,69,71]. 

Visual dysfunction caused by retinal degeneration, measured noninvasively by electroretinogram (ERG), showed an obvious reduction in the ERG response generated at the post-receptor level of the inner retina [50,51]. In a rodent model of ischemic glaucomatous injury, a functional impairment was indicated by a significantly smaller oscillatory potential amplitude and b-wave/a-wave ratio [50], the latter being a sensitive prognostic measure of ischemic injury in both animal models and humans [233,234,235,236]. Similar results were seen in NMDA-induced RGC injury, showing a significant reduction in the amplitude of the photopic negative response, which implied a post-receptor impairment [51]. However, treatment with lutein successfully restored the retinal function on ERG in both models of glaucomatous injury believed to contribute to optic neuropathy in POAG [50,51]. Additionally, lutein markedly enhanced visual function in vivo in excitotoxic-induced RGC injury, as shown by significant improvements in white–black discrimination during a visual behavior assay [51]. These findings provide further support to macular carotenoids’ nutraceutical effect in maintaining visual function against neurodegenerative insult.

However, it is worth noting that findings from in vitro studies evaluating the effect of supplementation with lutein and/or zeaxanthin using the disputed RGC-5 cell line [237,238,239] have been purposefully omitted from this review [240,241]. The original publication has since been redacted for erroneously characterizing the RGC-5 cell line to be of rat retinal ganglion origin [237,239]. It is now known to correspond to the mouse cell line 661W, which has been confirmed to be of cone photoreceptor origin [238,239,242,243,244]. 

While results from experimental models of glaucomatous injury indicate that the carotenoids lutein and/or zeaxanthin are sufficiently neuroprotective against neurodegeneration in the inner retina, there are some limitations to these findings that must be reviewed. Briefly, the absence of studies on the effects of carotenoid treatment in non-murine models restricts the translative potential for clinical use due to species differences between humans and rodents; specifically, the absence of the macula in these animals [245]. Further, the limited sample size may result from the complex nature of glaucoma as a multifactorial disease. This becomes clear as experimental models of glaucomatous RGC loss induced by transient ischemia–reperfusion through artificial IOP elevation [46,47], internal carotid artery occlusion [49,50], or chemically induced hypoxia [48,50] are incomplete models of the etiopathogenesis of glaucoma in humans [245]. Only one study evaluated the protective effects of lutein in a model of RGC injury induced by intravitreal NMDA injection [51]. However, one may conclude that there is substantial evidence in support of the anti-oxidative, anti-inflammatory, and pro-survival capacity of the carotenoids lutein and zeaxanthin in protecting against retinal neurodegeneration.

### 5.3. Carotenoids in the Managament of Glaucoma (Clinical Studies)

A growing body of evidence strongly suggests that a reduction in antioxidants resulting from prolonged oxidative stress is an essential driver in the initial sequence of interconnected mechanisms that contribute to the glaucomatous pathogenesis [23,24,25]. In the aqueous humor of patients with POAG, reports consistently demonstrate a significant reduction in total antioxidant capacity when compared with age-matched healthy controls [246,247,248]. One school of thought suggests that this response may be the longstanding consequence of protective mechanisms in the retina involving endogenous antioxidant defenses attempting to maintain redox homeostasis and limit oxidative injury by neutralizing pro-oxidant stressors [76,248,249]. Indeed, enhanced enzymatic activity of the potent antioxidants glutathione peroxidase and superoxide dismutase is seen in the glaucomatous eye [76,250,251] concomitantly with overexpressed markers of oxidation products (e.g., malondialdehyde) in the serum and aqueous humor [246,247,252,253]. Data from a meta-analysis corroborate these findings, providing consistent evidence of a systemic imbalance between oxidative stress and antioxidant levels in various types of glaucomatous injury, including but not limited to POAG [253]. However, cross-sectional studies indicate that the reduction in serum antioxidant status may be more profound in patients with open-angle glaucoma in comparison with closed-angle or pseudoexfoliation types [175]. Thus, it is clear that diminution of the endogenous antioxidant capacity, in response to persistent oxidative stressors in glaucomatous eyes, is an important pathogenetic mechanism in the occurrence and progression of POAG.

### 5.4. Dietary L/Z Intake and Risk of Glaucoma—Epidemiology Studies

To date, a number of epidemiological studies have evaluated the potential association between dietary carotenoid consumption (i.e., lutein and zeaxanthin, L/Z) and incident glaucoma, largely with inconsistent results [153,154,155,156,157]. From data collected by two large-scale prospective cohorts totaling over 100,000 participants, pooled multivariate analyses from the Nurses’ Health Study (NHS) and the Health Professionals Follow-up Study (HPFS) found that increased dietary levels of L/Z were the only serum antioxidants with material relevance to incident glaucoma [155]. Adjusting for the putative time delay from the etiologically relevant intake/exposure until the date of clinical diagnosis (i.e., a four-year lagged analysis), researchers found that individuals in the highest quintile of L/Z consumption had a significantly reduced risk for prevalent POAG than those in the lowest quintile (rate ratio (RR) = 0.68, 95% confidence interval (CI): 0.49–0.93) [155]. Conversely, a univariate analysis of antioxidant equivalents from a population-based cohort aged 55 years and older in the Rotterdam Study found no protective association between L/Z consumption and risk for open-angle glaucoma [157]. Despite this, an updated report from the NHS and HPFS found a 20% reduction in POAG incidence among individuals with higher daily servings of food groups rich in L/Z content (RR = 0.82, 95% CI: 0.69–0.97), more specifically green leafy vegetables such as kale, lettuce, and cooked/raw spinach [156,254,255]. However, these findings were suggested to largely be attributed to the greater intake of dietary nitrates derived from vegetable consumption [156,256,257]. After adjusting for dietary carotenoids and additional nutrients, a similar inverse association was seen between dietary nitrate consumption and risk of POAG (RR = 0.67, 95% CI: 0.52–0.85), which retained statistical significance [156]. Nonetheless, it remains unclear whether these effects are truly independent of the strongly associated nutrient content of lutein and zeaxanthin obtained simultaneously from these leafy green vegetable food groups.

Despite the preliminary evidence from large-scale cohort studies being inconsistent in substantiating a relationship between dietary carotenoids and glaucoma, evidence from other groups seems to corroborate a protective association with greater consumption of food groups rich in L/Z content [153,154]. One school of thought suggests that higher intake of L/Z may serve to improve the retinal vasculature’s caliber and protect against signs of vascular dysregulation [258]. Established risk factors of cardiovascular disease are strongly associated with glaucomatous RNFL atrophy and POAG incidence [256,259,260,261]. A cross-sectional analysis from one report in the Study of Osteoporotic Fractures found a significant protective association among women aged 65 and older who consumed ≥1 serving of collard greens and kale per month (odds ratio (OR) = 0.31, 95% CI: 0.11–0.91) [153]. Several common leafy green vegetables, including kale and collard greens, from the cabbage species *Brassica oleracea* are major dietary sources of L/Z [123,254,262,263]. More recently, a cross-sectional analysis from a cohort of African-American women included in the Study of Osteoporotic Fractures found a more significant protective trend against incident glaucoma among women consuming more collard greens/kale on a weekly basis (≥1 serving per week; OR = 0.43, 95% CI: 0.21–0.85) [154]. In fact, after adjusting for potential confounders, researchers found that African-American women in the highest quartile of daily L/Z intake (≥4000 µg/day) were strongly associated with a significant reduction in the odds of glaucoma (OR = 0.43, 95% CI: 0.21–0.88) [154]. Moreover, these results may be attributable, at least in part, to an increase in daily servings of L/Z, which seem to reflect a protective trend in retinal vasculature against glaucomatous etiology.

The evidence from epidemiological studies investigating the association between dietary L/Z intake and risk of incident glaucoma is promising, but not without certain limitations [153,154,155,156,157]. An inherent challenge in studying glaucoma prevalence in large-scale nutritional epidemiology remains the insidious nature of this neurodegenerative optic neuropathy [78,153,154,264]. Hence, the etiologically relevant time period/exposure likely involves dietary behaviors that occurred several years prior to the date of diagnosis in consequence of clinical signs becoming apparent only after substantial damage is incurred to the ONH [78,264]. Variation among the inclusion criteria for the presence of glaucoma in large-cohort studies, relying largely on self-reported cases, may limit the interpretability of these results for the general population [155,156,157]. Meanwhile, cross-sectional cohorts were ascertained by optic nerve imaging and VF assessment during a clinical eye examination [153,154]. None of the aforementioned epidemiology studies directly investigated the association between serum levels of carotenoids and risk of POAG. However, the protective trends demonstrated herein provide support to greater dietary intake of the carotenoids L/Z among older individuals and those at risk of ocular diseases, including glaucoma.

### 5.5. Macular Pigment Optical Density and Primary Open-Angle Glaucoma

The neuroprotective potential of macular pigment concentrations of L/Z in the pathogenesis of glaucomatous optic neuropathy have been explored in a limited number of studies. To date, there have been six clinical studies that explicitly measured MPOD levels in glaucomatous eyes to determine the relationship between macular pigment status and the presence of POAG [54,55,56,57,58,59]. Although some discrepancies exist among reports, the evidence generally suggests that MPOD levels are significantly reduced in glaucomatous eyes and may be further compromised with greater disease severity [56,57,58,59]. Similarly, there is a need to substantiate the structure–function relationship between macular ganglion cell thinning and the extent of functional visual loss associated with glaucoma [265,266,267]. A summary of clinical studies in relation to the putative association between MPOD and clinical parameters of POAG is given in Table 2.

Glaucomatous neurodegeneration is multifaceted and has a complex etiopathogenesis, so it is not surprising that there are differing schools of thought regarding possible causative mechanisms whereby the MPOD may be lower in patients with POAG. Several prospective studies found significant reductions in the spatial profile of macular pigments within 1° of retinal eccentricity in glaucomatous eyes using a customized HFP (cHFP) technique [56,58,59]. Similarly, the MPOD was also significantly reduced in a cohort of Chinese patients with POAG (*p* < 0.001) as compared with age-matched controls and measured by single-wavelength reflectometry [57]. Researchers suggest that reductions in MPOD may be explained, in part, by apoptosis of RGCs and neurodegeneration of the nerve fiber layer as concomitants of the pro-oxidative environment in glaucomatous retina. Thus, the loss of photoreceptors and ganglion cells may severely compromise the localization of macular pigment within the innermost retinal layers of the fovea [59,264,268,269]. In fact, cross-sectional studies observed, upon FD-OCT imaging of the retina, that the MPOD was significantly lower in those who exhibited structural thinning of the ganglion cell complex (GCC) that encroaches upon the foveal zone [58,59]. Data from these studies determined that the presence of foveal GCC loss was a major determinant of MPOD levels in glaucomatous eyes [58,59]. Similar results from a case-control study mirrored these findings, demonstrating that both the maximum and mean MPOD levels displayed a positive relationship with macular GCC thickness on SD-OCT (*p* < 0.001 and *p* = 0.001, respectively) [57]. Glaucomatous eyes with foveal GCC loss also exhibited greater disease severity, as evidenced by thinning of the RNFL and the GCC layer, an increased cup-to-disc ratio, or narrowing of the neuroretinal rim (*p* < 0.001, for all) [58]. This is of relevance because early glaucomatous injury to retinal substructures likely involves the macula, in particular the layers that comprise the GCC [99,267]; that is, the nerve fiber layer, the ganglion cell layer, and the inner plexiform layer, which correspond to the axons, cell bodies, and dendrites of retinal ganglion cells, respectively. Thus, given that glaucoma appears to target the retinal layers in which macular pigment is primarily localized, this may explain, at least in part, the finding that the MPOD appears to be lower in more severe cases of POAG with foveal involvement.

Macular involvement with corresponding VF defects in early stages of glaucoma may be more common than previously realized, as evidenced by paracentral scotoma on standard automated perimetry [270,271]. Thus, glaucomatous loss of foveal RGCs with a subsequent MPOD decrease is likely to be of significance in relation to vision-related quality of life for patients with POAG. The literature provides a strong rationale to support a structure–function relationship between a greater reduction in MPOD with foveal ganglion cell loss and corresponding VF defects in glaucoma [37,57,58,59,99,270]. A cross-sectional analysis found that functional vision loss in the central retina (within 10° of fixation) was significantly correlated with a lower MPOD at multiple retinal eccentricities among those with foveal GCC involvement (*p* < 0.01 at 0.25°, 0.5°; *p* = 0.01 at 1°) [59]. Glaucomatous foveal scotoma was assessed by mean deviation (MD) on the Humphrey 10-2 VF test because it samples macular visual function more precisely than the standard 24-2 VF test [59,272,273,274,275]. Furthermore, not only were lower MPOD levels associated with greater glare-related disability and reduced glare-related visual performance in the glaucomatous eye, but it appeared that this relationship was mediated by foveal involvement [59]. Given that the macula contains the highest density of RGCs (~50% concentrated within 4.5 mm of the fovea) [34,276], observations in which foveal involvement relates to a lower MPOD, as encountered in glaucoma, may therefore elucidate, at least in part, the degree of functional vision loss in cases of POAG.

On the other hand, two prospective studies were unable to demonstrate a significant correlation between macular pigment and the presence of POAG using dual-wavelength autofluorescence imaging [54,55]. In the first study, a cross-sectional analysis found that macular pigment volumes were comparable between glaucomatous eyes and controls [55]. Similarly, a case-control study found no significant evidence that the MPOD was lower in glaucoma patients, or that a lower MPOD was linked to the presence of glare symptoms [54]. The inconsistency in results may be attributed, at least in part, to differences in the study methodology with regard to the MPOD measurement technique [132,133,134]. Briefly, because the cHFP technique is psychophysical in nature, it cannot be ruled out whether the presence of glaucoma may have influenced the individual’s fixation capacity and affected the acquisition of MPOD measurements. Despite this, cHFP remains the current gold standard approach to MPOD measurement in clinical applications.

The results from these [54,55,56,57,58,59] clinical studies that have investigated the relationship between MPOD and glaucomatous neurodegeneration are promising, but not without several limitations: (1) with one exception [57], the inclusion criteria for the presence of glaucoma were not exclusive to individuals with POAG; (2) with one exception [58], foveal involvement was analyzed homogenously among different subtypes of open-angle glaucoma in relation to MPOD levels; (3) there were relatively small and unequal sample sizes based on ocular health status (glaucoma vs control) in several studies; and (4) all studies were observational in nature only. Additional research is necessary to further investigate the potential relationship whereby the MPOD appears to be lower in glaucomatous eyes and may be further compromised among those with greater disease severity.

### 5.6. Carotenoid Supplementation—Interventional Studies

Prospective studies in patients with glaucoma evaluating the efficacy of dietary carotenoid supplementation with antioxidant micronutrient formulas in randomized controlled trials (RCTs) remain limited and controversial at best (Table 3) [60,61,62]. In an open-label long-term follow up assignment among patients with mild/moderate POAG, two experimental antioxidant formulas containing lutein (6 mg) and zeaxanthin (0.3–0.5 mg), with or without omega-3 fatty acids, failed to demonstrate any difference between groups at the end of trial period [60]. Researchers found no significant change with either supplement after a two-year follow up on visual field loss or quantitative measures indicating thinning of the RNFL or the GCC upon FD-OCT scanning [60]. In contrast, a prospective study with an antioxidant nutraceutical formula containing lutein (10 mg) and zeaxanthin (2 mg) in equal groups of healthy controls and POAG patients found a significant protective trend with carotenoid supplementation after a six-month follow up [62]. In the POAG group, a marked increase in plasma antioxidant capacity (*p* = 0.028), with a concomitant reduction in the pro-oxidant stressor malondialdehyde, reached greater significance (*p* = 0.005) [62]. Similar protective trends have also been observed in clinical trials with a carotenoid supplement nutraceutical conducted in patients with pseudoexfoliative glaucoma [61]. However, there is simply a paucity of evidence from clinical trials to sufficiently determine the precise neuroprotective potential in open-angle glaucomatous injury. Hence, further interventional studies are required should carotenoid vitamin therapy be immediately considered a primary form of treatment in the management of glaucomatous neurodegeneration.

Inconsistency among findings from these intervention trials may be attributed, at least in part, to a variety of potential factors and limitations. Most notably, none of the available trials included a serum analysis of lutein and zeaxanthin or measured MPOD levels within the study design. Limitations regarding low absorption rates continue to pose a major challenge to clinical trials [277,278]. A growing body of evidence strongly suggests that conventional delivery systems, such as the soft-gel capsules used within all three reports, may significantly limit the bioavailability of carotenoids and consequently impede the desired health benefits in the retina [43,122,277,278,279,280]. To overcome such a limitation, greater amounts of carotenoids may be necessary along with a longer duration of intake. Assimilation and transport of carotenoids from food matrices are also heavily influenced by anthropometric features as well as gender, ethnicity, and age [45,116,279,281,282,283,284,285,286]. Enhanced performance in the bioavailability and accumulation of lutein and zeaxanthin in the retina has been demonstrated using micronized and nanoemulsion-based microsphere techniques [43,278,286,287,288,289]. Moreover, clinical implementation of these carotenoid-containing microspheres has shown improved dissolution efficacy while maintaining overall safety when applied intraocularly.

## 6. Conclusions

There is a theoretical rationale for the use of carotenoid vitamin therapy as an adjunctive treatment in the management of glaucoma. It appears that the glaucomatous retina creates an environment hostile to the survival of neurons, in part due to sustained oxidative injury with a concomitant depletion of endogenous antioxidant defenses. The cumulative effect of glaucoma appears to compromise the localization of the macular carotenoids lutein, zeaxanthin, and *meso*-zeaxanthin, contributing to deteriorating retinal health and ultimately irreversible vision loss. Based on the available preliminary results, carotenoid vitamin therapy has shown promise in augmenting MPOD levels and enhancing visual performance. In this regard, there is sufficient evidence from preclinical studies to support the synergic neuroprotective benefits of carotenoid supplementation as an adjunctive nutraceutical approach to the management of glaucoma. However, the available results from clinical trials are largely controversial and insufficient at present, thereby warranting further prospective controlled studies. Thus, it remains to be seen if this adjunctive nutraceutical approach, in combination with IOP-lowering therapy, can provide additional protective benefits to glaucomatous eyes.

## Figures and Tables

**Table 1:** Preclinical studies evaluating the effect of the carotenoids lutein and/or zeaxanthin on glaucomatous/RGC injury.

Author, Year	Glaucoma/RGC Injury Model	Intervention	Main Findings
Choi, 2006 [46]	Retinal I/R injury by artificial IOP elevation	Lutein	Prevented an increase in nNOS and COX-2 expression following ischemic injury
Dilsiz, 2006 [47]	Retinal I/R injury by artificial IOP elevation	Lutein	Reduced lipid peroxidation and activation of caspase-3 and improved GSH levels
Fung, 2016 [48]	Retinal I/R injury by chemically induced hypoxia in rMC-1 cells	Lutein	Improved glial cell survival and viability following hypoxic injury through modulating apoptosis and autophagy
Li, 2009 [49]	Retinal I/R injury by middle cerebral artery occlusion	Lutein	Enhanced ganglion cell survival, viability, and morphology following I/R injury
Li, 2012 [50]	Retinal I/R injury by middle cerebral artery occlusion and chemically induced hypoxia in rMC-1 cells	Lutein	Improved measures of retinal function, with reduced gliosis and increased cell survival
Zhang, 2016 [51]	RGC injury by intravitreal NMDA injection	Lutein	Augmented ganglion cell viability with improved retinal function parameters

Abbreviations: RGC, retinal ganglion cells; I/R, ischemia–reperfusion; IOP, intraocular pressure; nNOS, neuronal nitric oxide synthase; COX-2, cyclooxygenase-2; GSH, glutathione; rMC-1, rat Muller glial cells; NMDA, N-methyl-d-aspartic acid.

**Table 2:** Clinical studies evaluating macular pigment optical density (MPOD) in open-angle glaucoma.

Author (Year)	Participants	MPOD Measurement Technique	Exposure Variable	Main Findings
Bruns (2020) [54]	33 POAG cases 43 healthy controls	Dual-wavelength AFI	0.51°, 1.02°, and 1.99°	No evidence of lower MPOD in glaucomatous eyes
Daga (2018) [55]	85 POAG cases 22 healthy controls	Dual-wavelength AFI	MP volume over 7°	MP volume was comparable between glaucomatous eyes and controls
Igras (2013) [56]	36 POAG cases 54 healthy controls	HFP	0.5°	Lower MPOD in glaucomatous eyes compared with controls (*p* = 0.03)
Ji (2016) [57]	30 POAG cases 52 healthy controls	Single-wavelength Reflectometry	MPOD mean over 7°	Significantly reduced MPOD and GCC thickness in POAG patients (*p* < 0.001, for all)
Siah (2015) [58]	44 POAG cases (22 with foveal involvement)	cHFP	0.25°, 0.5°, and 1°	Glaucomatous eyes with foveal GCC loss had a lower overall MPOD (*p* < 0.001, for all)
Siah (2018) [59]	88 OAG cases	cHFP	0.25°, 0.5°, and 1°	Lower MPOD was correlated with the magnitude of the central 10° field loss (*p* < 0.01, for all)

Abbreviations: MPOD, macular pigment optical density; POAG, primary open-angle glaucoma; AFI, autofluorescence imaging; MP, macular pigment; HFP, heterochromatic flicker photometry; GCC, ganglion cell complex; cHFP, customized heterochromatic flicker photometry; OAG, open-angle glaucoma.

**Table 3:** Characteristics of the eligible randomized clinical trials.

Author (Year)	Participants	Duration	No. of Groups	Interventions	Treatment Schedule
Garcia-Medina (2015) [60]	117 patients with mild/moderate POAG, aged (61.5 ± 11.7) years	2 years	3	6 mg L and 0.3 mg Z (multivitamin plus ω-3); 6 mg L and 0.5 mg Z (multivitamin only); placebo	5 days/wk
Romeo Villadóniga (2018) [61]	47 patients with PEX, aged (70.3 ± 5.0) years	6 months	2	10 mg L and 1 mg Z (multivitamin); placebo	Daily
Sanz-González (2020) [62]	15 patients with POAG and 15 controls, aged 40–75 years	6 months	2	10 mg L and 2 mg Z (multivitamin)	Daily

Abbreviations: POAG, primary open-angle glaucoma; L, lutein; Z, zeaxanthin; ω-3, omega-3 fatty acids; PEX, pseudoexfoliative glaucoma.
